# Effect of Low FODMAPs Diet on Irritable Bowel Syndromes: A Systematic Review and Meta-Analysis of Clinical Trials

**DOI:** 10.3390/nu13072460

**Published:** 2021-07-19

**Authors:** Jongsung Hahn, Jeongwon Choi, Min Jung Chang

**Affiliations:** 1 Department of Pharmacy and Yonsei Institute of Pharmaceutical Sciences, College of Pharmacy, Yonsei University, Incheon 21983, Korea; jshahn@yonsei.ac.kr; 2 Department of Industrial Pharmaceutical Science, College of Pharmacy, Yonsei University, Incheon 21983, Korea; qwe862@hanmail.net

**Keywords:** irritable bowel syndrome, IBS, low FODMAP, diet, meta-analysis

## Abstract

We conducted a meta-analysis exploring the effect of a low fermentable oligo-, di-, monosaccharides, and polyols diet (LFD) on the overall symptoms, quality of life, and stool habits of irritable bowel syndrome (IBS) patients. The meta-analysis was performed using a random-effects method. The effect size was presented as weighted standardized mean difference (SMD) and 95% confidence interval (CI). Subgroup analyses were conducted to determine the potential effects of covariates on the outcome. Twenty-two papers were included. The LFD group showed a moderate reduction in symptom severity and a slight improvement in quality of life compared to the control group (SMD, −0.53 and 0.24; 95% CI, −0.68, −0.38 and 0.02, 0.47, respectively). IBS symptom improvement was consistent between subgroups stratified according to proportions of female patients, study durations, IBS subtypes, assessment methods, and control interventions. Three studies regarding stool habits change in IBS-D patients showed a significant decrease in stool frequency (mean differences [MD], −5.56/week; 95% CI, −7.40, −3.72) and a significant improvement in stool consistency (MD, −0.86; 95% CI, −1.52, −0.19) in the LFD group compared to the control group. This is the most updated meta-analysis including studies that adopted diverse control interventions such as dietary interventions, supplementation, habitual diets, and lifestyle changes.

## 1. Introduction

Irritable bowel syndrome (IBS) is a chronic functional gastrointestinal disorder characterized by changes in bowel habits accompanied by abdominal pain [1]. Its estimated prevalence in the general population ranges from 5% to 20% [1,2,3], with a higher prevalence in females than in males (12.0%; 95% confidence interval [CI], 9.3–15.0 vs. 8.6%; 95% CI, 6.3–11.2; odds ratio [OR], 1.46; 95% CI, 1.33–1.59) [4]. The chronic nature of IBS with recurrent and exacerbating symptoms negatively impacts quality of life (QoL), work productivity, and social functioning [5]. The pathophysiology of IBS is multifactorial and heterogeneous, which includes disordered communication between the gut and brain, intestinal dysmotility, low-grade inflammation, visceral hypersensitivity, alterations in the gut microenvironment, genetic predisposition, stress, and dietary changes [6].

Traditionally, treatment of IBS focused on pharmacological medications such as laxatives, antispasmodics, and antidepressants. In recent years, however, attention has been given to dietary interventions and cognitive/emotional therapies [7,8]. Diets low in fermentable oligo-, di-, monosaccharides, and polyols (FODMAPs) have gained importance and have been proposed as a first-line dietary therapy in IBS management [9].

Recently, Lanen et al. found that a low-FODMAP diet (LFD) reduced IBS severity to a moderate-to-large extent and increased IBS-QoL scores when compared with a control diet in a meta-analysis (*n* = 12) [10]. However, their study limited the control interventions to traditional IBS diet (sham diet, balanced Mediterranean diet, National Institute for Health and Care Excellence (NICE) guideline diet, and British Dietetic Association (BDA) guideline diet) or habitual diet or high FODMAP diet. Therefore, the purpose of this study was to provide an updated meta-analysis that compared an LFD group with a control group that was given not only the traditional IBS, habitual, and high FODMAP diets but also structured dietary interventions (such as low-lactose diet, gluten-free diet), supplementation (prebiotics, probiotics), and lifestyle changes (yoga, hypnotherapy) for symptom reduction and improvement of quality of life. We also explored the LFD effect on stool habits (frequency and consistency).

## 2. Materials and Methods

The search strategy, study selection, data extraction, and data analysis were conducted in agreement with the Preferred Reporting Items for Systematic reviews and Meta-analysis (PRISMA) statement [11].

### 2.1. Data Sources and Search Strategy

We searched the Cochrane Central Register of Controlled Trials (CENTRAL), PubMed, and Embase for relevant studies up to February 2021 using the variations and combinations of the following search terms: IBS, irritable bowel syndrome, irritable bowel, irritable colon, LFD, low FODMAP diet, diet restriction, FODMAP, fermentable oligosaccharides disaccharides monosaccharides and polyols, fermentable, short-chain carbohydrate, oligo-saccharide, di-saccharide, mono-saccharide, polyol, fructooligosaccharide, galactooligosaccharide, fructan, fructose, galactan, galactose, lactose, sorbitol, mannitol, xylitol, maltitol, sweetener, sweetening agent. There were no language restrictions.

### 2.2. Study Selection (Inclusion and Exclusion Criteria)

The studies had to comply with the following inclusion criteria: include an adult population (older than 18 years) diagnosed with some type of IBS; study the effect of LFD on IBS symptoms, quality of life, and stool habit and compare it with that of a control diet or intervention; and be clinical trials. We included all RCTs with a parallel, cross-over, or factorial design; Intervention and observational studies were also included.

The following studies were excluded: conference abstracts, non-comparative studies, studies with no comparators or insufficient data provided to calculate effect sizes, studies that included children, and studies based on secondary sources. If the same study was reported in more than one publication, we included the article with the longest follow-up to avoid duplication of information.

Two researchers (JH and MJC) independently screened the articles by title and abstract after removing duplicates. The obtained articles were subjected to a full-text evaluation according to the inclusion criteria. Any disagreements were resolved by discussion between the two investigators.

### 2.3. Data Extraction and Quality Assessment

The general information of the study (author, publication year, country, and journal), study details (study design, sample size, study duration, types of intervention, and types of control), population characteristics (age, sex, IBS diagnostic criteria, and IBS subtype), and outcomes (IBS symptom reduction, IBS-QoL, and stool frequency, and consistency) were retrieved independently by two researchers (JH and MJC).

The primary outcome of interest was IBS symptom reduction assessed using the IBS- Severity Scoring System (IBS-SSS). It consists of five questions that measure abdominal pain severity, abdominal pain frequency, abdominal bloating, bowel habit dissatisfaction, and interference with quality of life on a 0–100 mm visual analog scale (VAS), with total scores ranging from 0 to 500. Based on the IBS-SSS scores, severity was classified as mild (<150), moderate (150–300), or severe (>300) [12]. Studies using other scores of overall symptom severity, such as VAS scores and Likert scale scores, were also included. 

The co-primary outcome was clinical improvement in IBS symptoms, as a proportion of responders, according to a binary assessment using the cutoff of reduction in IBS-SSS > 50 or a positive response to an IBS adequate relief question (Did you have adequate relief of IBS-related abdominal pain or discomfort?) [13]. 

The secondary outcomes (when available) were as follows:

1. Health-related quality of life as measured by IBS-QoL questionnaire, which consists of 34 IBS specific items with eight subscales: dysphoria, interference with activities, body image, health worry, food avoidance, social reaction, sexual function, and relationships. The scores are averaged and transformed to a 0–100 scale, with increasing scores indicating a better quality of life [14].
2. Stool consistency was assessed by the Bristol Stool Form Scale (BSFS). The BSFS is an ordinal scale of stool types, ranging from the hardest (Type 1) to the softest (Type 7) [15]. The stool frequency was converted to per week frequency in all the included studies.

All available outcomes’ means and standard deviations at baseline and endpoints of both intervention and control groups were combined and the standard mean difference values were calculated by using Hedges’ adjusted *g*. If the means and standard deviations were not reported in the text, the data were extracted from the table or graph (using a program for digitizing graphs and plots) [http://getdata-graph-digitizer.com/. Accessed 1 June 2021]. When the study included multiple groups, a combined control group was compared to the LFD group.

The methodological quality of the included studies was assessed by two independent researchers (JH and MJC) using the Cochrane risk of bias tool [16]. According to the guidelines, the overall quality of each study was considered good (low risk for more than two items), fair (low risk for two items), or weak (low risk for less than two items).

### 2.4. Data Analysis

All meta-analyses were conducted using R software (version 4.0.5) with the general package for meta-analysis version 4.18-0. In the present study, the effect size was based on the mean difference (MD), standardized mean difference (SMD), and weighted MD or SMD, along with its 95% CI. For dichotomous outcomes, we calculated the OR with 95% CI. The threshold for statistical significance was set at *p* < 0.05. Heterogeneity was estimated using a Chi-square test on Cochrane’s Q statistic and quantified with the I^2^ statistic value, which was interpreted to signify low heterogeneity at ≤25%; moderate heterogeneity at 26–50%; and high heterogeneity at >50% [17]. A random-effects model with inverse-variance weighting was used. 

Subgroup analyses were conducted to determine the potential effects of the following covariates on the outcome: IBS subtype (including all types, only diarrhea-predominant IBS [IBS-D] type, or excluding constipation-predominant IBS [IBS-C] type), female proportion (>75% (median) or <75%), duration of the intervention (1−2 days, 3−4 weeks, or 6−12 weeks), outcome assessment method (IBS-SSS or other) and control group (dietary intervention, habitual diet, high FODMAP, or other treatment (supplementation and lifestyle changes)). 

We constructed a funnel plot and used an Egger’s test to assess the possibility of publication bias. When 10 or more studies were available, *p* < 0.05 was considered indicative of statistically significant publication bias [18]. We also performed sensitivity analyses to assess the robustness of the results. 

## 3. Results

### 3.1. Study Description

**Figure 1:**
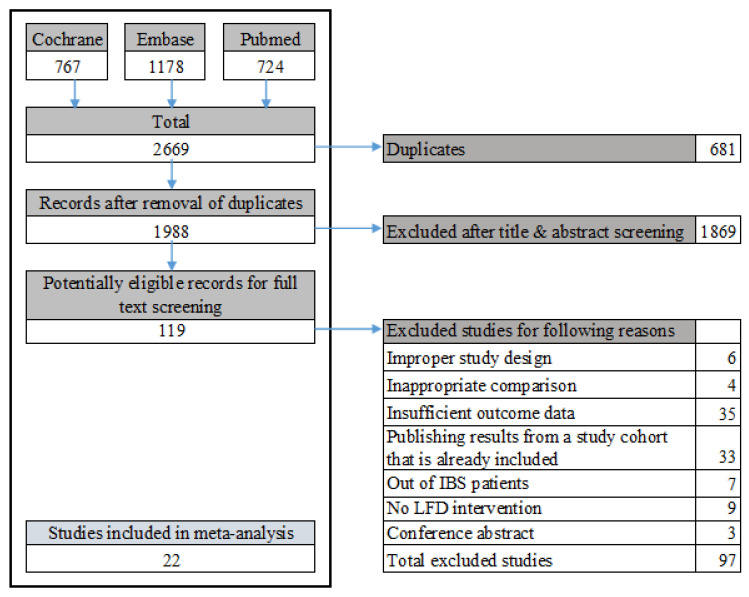
Identification and selection of the studies in the databases. LFD, low fermentable oligo-, di-, and monosaccharide and polyol diet.

A total of 2669 publications were identified using a predefined search strategy. After duplication-checking, titles and abstracts of 1988 records were screened, leading to the full-text assessment of 119 studies. After the exclusion of 97 studies, only 22 studies reporting IBS symptoms or quality of life outcomes were included in the meta-analysis (Figure 1 and Table 1). All 22 studies were published between 2010 and 2021. Eleven studies compared LFD with other dietary interventions (low-lactose diet, gluten-free diet, sham diet, BDA, and NICE guideline diet); five with a habitual diet (including regular rye bread and no dietary education); two with supplements (including probiotic and prebiotic); two with other lifestyle changes (including yoga and gut-directed hypnotherapy); and two with a high-FODMAP diet. Regarding designs, 20 were RCTs (90.9%) and two were non-RCTs (9.1%). One study was only conducted on females; others were conducted on both genders. Additionally, four studies showed results specifically referred to IBS-D patients, five studies had results on exclusion of IBS-C type patients, and others had results on all types of patients, categorized according to the Rome criteria. Of all studies reviewed, 21 and 11 investigated IBS symptoms and quality of life, respectively. Regarding the reporting of symptoms, 13 studies used the IBS-SSS form, seven studies used the VAS or Likert scale form, and the rest only reported response to an IBS adequate relief question. 

### 3.2. Results of Meta-Analysis

#### 3.2.1. Primary Outcome

**Figure 2:**
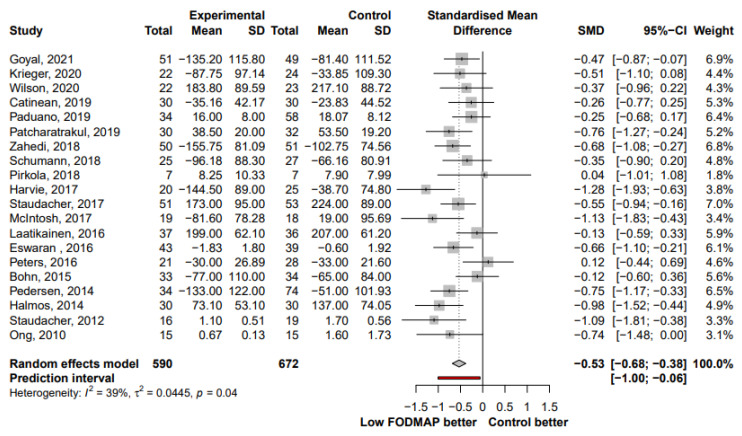
Forest plot showing standardized mean differences for IBS symptom severity reduction. SD, standard deviation; SMD, standardized mean difference; CI, confidence interval.

**Figure 3:**
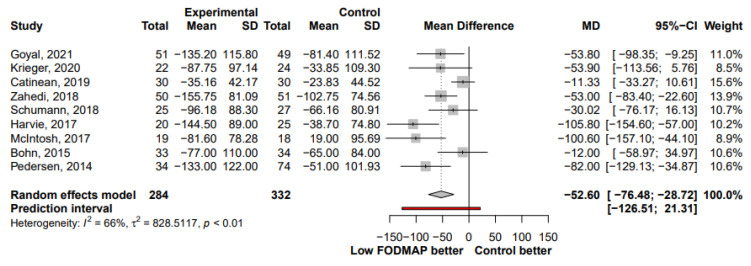
Forest plot showing mean IBS-SSS reduction in studies that used pre- and post-intervention IBS-SSS measures. SD, standard deviation; MD, mean difference; CI, confidence interval.

**Figure 4:**
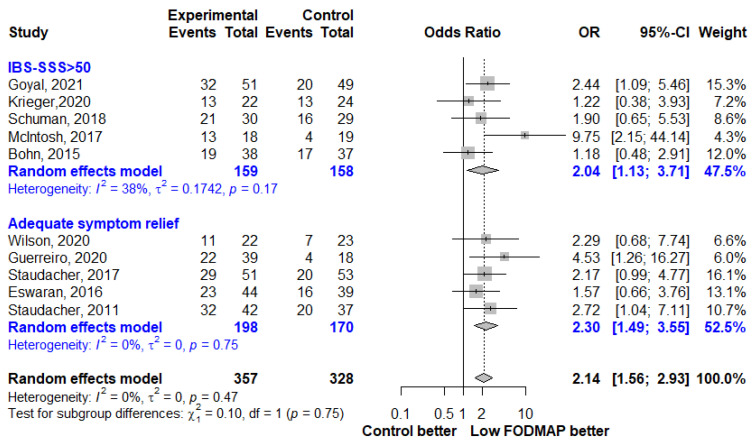
Forest plot showing odds ratios for clinical improvement in IBS symptoms as a proportion of responders based on reduction in IBS-SSS > 50 or a positive response to an IBS adequate symptom relief question. OR, odds ratio; CI, confidence interval.

The effect of LFD on IBS symptom severity was reported in 20 studies. The LFD group showed a moderate reduction in IBS symptom severity compared to the control group (SMD, −0.53; 95% CI, −0.68, −0.38; I^2^ = 39%) (Figure 2). When analyzing studies that reported both pre- and post-intervention IBS-SSS, a mean reduction of 52.6 points (95% CI, −76.48, −28.72; I^2^ = 66%) was observed in the LFD group (Figure 3). The number of patients who showed a reduction in IBS-SSS of more than 50 points and who reported adequate symptom relief in the LFD group compared with the controls group is presented in Figure 4. A greater improvement in symptoms was seen in the LFD group than in the control group (OR, 2.14; 95% CI, 1.56, 2.93; I^2^ = 0%).

**Figure 5:**
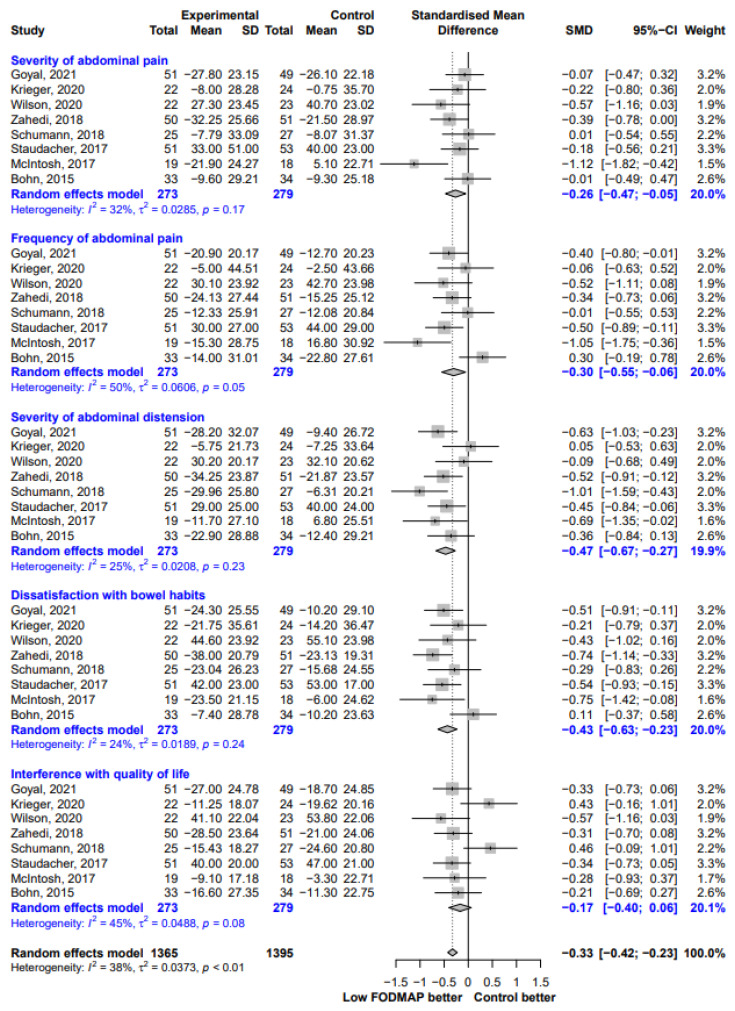
Forest plot showing standardized mean differences for five IBS-SSS subscale scores. SD, standard deviation; SMD, standardized mean difference; CI, confidence interval.

In the IBS-SSS subscale score analysis, the most substantial improvement occurred for “severity of abdominal distension” (SMD, −0.47; 95% CI −0..67, −0.27; I^2^ = 25%), followed by “dissatisfaction with bowel habits” (SMD, −0.43; 95% CI, −0.63, −0.23; I^2^ = 24%), “frequency of abdominal pain” (SMD, −0.30; 95% CI, −0.55, −0.06; I^2^ = 50%), and “severity of abdominal pain” (SMD, −0.26; 95% CI −0.47, −0.05; I^2^ = 32%). However, we could not find any significant difference in scores for “interference with quality of life” between the two groups (SMD, −0.17; 95% CI, −0.40, 0.06; I^2^ = 45%) (Figure 5).

#### 3.2.2. Subgroup Analysis

We conducted subgroup analyses to identify any covariates for between-study heterogeneity (Table 2). In all subgroups according to proportion of female participants, IBS subtype, study duration, symptom assessment method and control group type, the improvement in IBS symptom severity remained statistically significant (Figures S1–S5). 

#### 3.2.3. Secondary Outcomes

**Figure 6:**
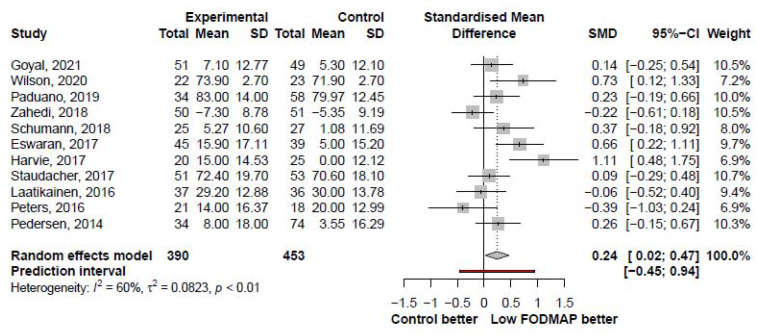
Forest plot showing standardized mean differences in IBS-QoL. SD, standard deviation; SMD, standardized mean difference; CI, confidence interval.

The IBS-QoL was analyzed in 11 studies and showed a slight improvement after LFD when compared with that after the control intervention (SMD, 0.24; 95% CI, 0.02, 0.47; I^2^ = 60%; Figure 6). IBS-QoL subscale score analysis was presented in Figure S6.

**Figure 7:**
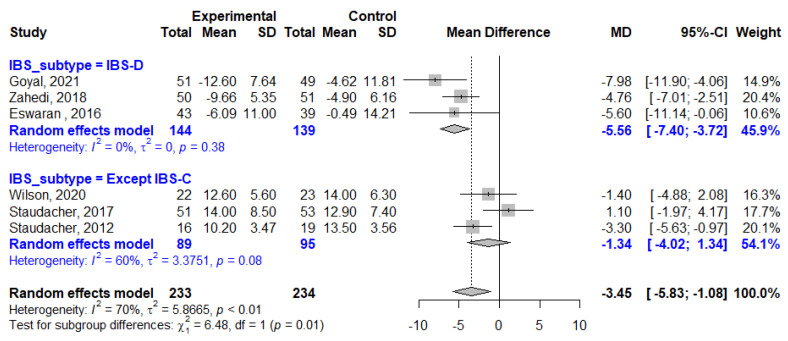
Forest plot showing mean differences in average weekly stool frequency. SD, standard deviation; MD, mean difference; CI, confidence interval.

**Figure 8:**
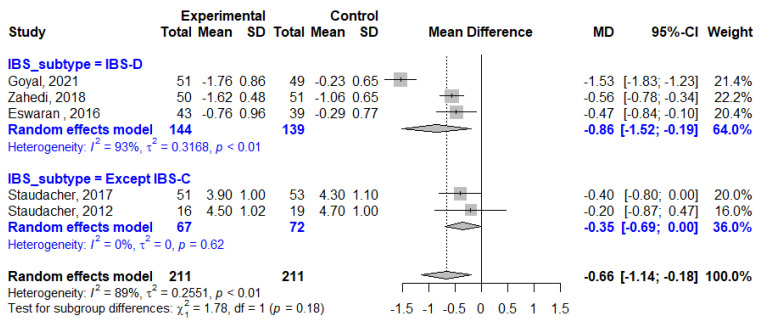
Forest plot showing mean differences in BSFS. SD, standard deviation; MD, mean difference; CI, confidence interval.

The average weekly stool frequency was reported in six studies, three study of which enrolled patients with IBS-D only and others included patients with any type of IBS except IBS-C. Overall, stool frequency was reduced by 3.45 per week in LFD patients compared to that in the control patients (MD, −3.45; 95% CI, −5.83, −1.08; I^2^ = 70%). The impact of LFD on stool frequency reduction in patients with only IBS-D (MD, −5.56; 95% CI, −7.40, −3.72; I^2^ = 0%) was greater than that on the whole cohort (Figure 7). The mean BSFS scores improved by −0.86 points in the LFD group compared to that in the control group in studies that involved only patients with IBS-D (MD, −0.86; 95% CI −1.52, −0.19; I^2^ = 93%, Figure 8).

### 3.3. Sensitivity Analysis, Publication Bias and Risk of Bias

**Figure 9:**
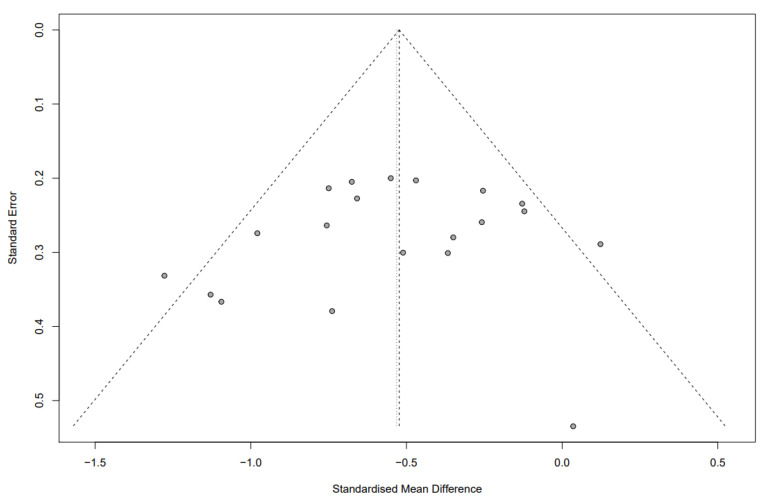
Funnel plot of studies that measured IBS symptom severity.

**Figure 10:**
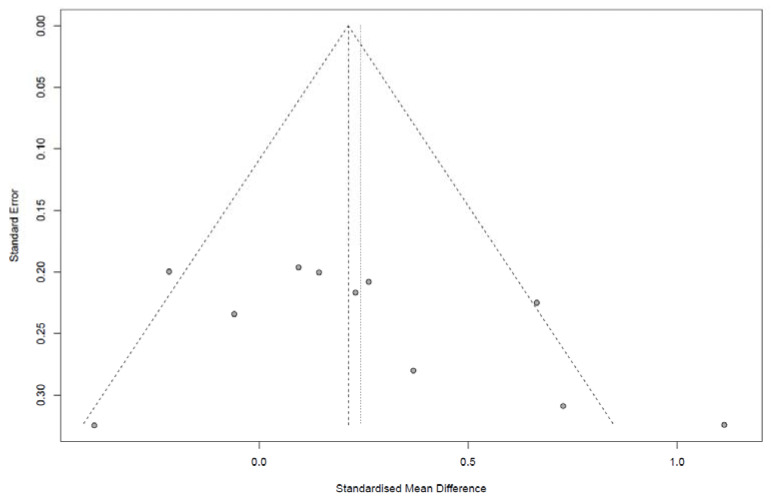
Funnel plot of studies that measured IBS related quality of life.

Sensitivity analysis for the outcomes of IBS symptoms and quality of life, conducted by omitting every study from the meta-analysis, are shown in Tables S1 and S2, respectively, and did not show a significant effect on the overall effect size. Overall, all included studies had some risk of bias, most showed unclear allocation concealment and high risk of bias in blinding of participants and personnel and outcome assessment (Table 3). In all, 18 studies (81.8%) were of good quality. Visual inspection of the funnel plot showed relatively symmetrical distribution, indicating the absence of publication bias (Figure 9 and Figure 10 for IBS symptom severity and quality of life, respectively). It was confirmed by Egger’s test, which did not show any significant statistical bias (*p* = 0.5482 and 0.2306, respectively).

## 4. Discussion

This is the most updated meta-analysis including the analysis of IBS-SSS subscale score and bowel habits. The most recent meta-analyses [10] included 12 studies and six studies, respectively, that assessed the effect of LFD on IBS symptoms and quality of life. We updated this meta-analysis by adding four newly published studies, as well as studies that adopted control groups subjected to different structured dietary interventions (low-lactose diet and gluten-free diet), supplementation (prebiotics and probiotics), lifestyle changes (yoga and hypnotherapy), and regular rye bread. To our knowledge, this is the first systematic review and meta-analysis to estimate the effect size of LFD in the treatment of IBS when compared to diverse control interventions separately. 

Our study found that LFD showed a moderate reduction in IBS symptom severity compared to the control interventions (SMD, −0.53; 95% CI, −0.68, −0.38), which is in line with the previous meta-analyses [10]. A multitude of different assessment methods are currently used to assess symptom severity in patients with IBS. Of these, the validated IBS-SSS is the broadest assessment tool for symptom-related aspects, and the VAS or Likert scale are simple methods to measure overall symptoms or pain [41]. The improvements in IBS symptoms were consistent between the subgroups of symptom assessment methods and showed only lower heterogeneity in the IBS-SSS subgroup than in the others (VAS or Likert scale subgroups) (SMD, −0.49 vs. −0.62; I^2^, 34% vs. 50%; *p* for between-subgroup heterogeneity, 0.51). When analyzing studies that reported both pre- and post-intervention IBS-SSS, a mean reduction in IBS-SSS of 52.6 points (95% CI, −76.48, −28.72) was found in the LFD group, which could be interpreted as reliably adequately improved [12].

Given that patients’ baseline IBS-SSS scores differed within and between studies, it was appropriate to define a 50-point reduction in IBS-SSS, which was associated with a clinically meaningful improvement [12], as a binary response and derive the response rate to evaluate the efficacy of LFD. The response to IBS adequate relief questionnaire, another binary endpoint, is also the best choice for assessing global symptoms and has been used frequently [13]. Therefore, we conducted a meta-analysis using these two binary endpoints of IBS symptoms, and found a greater improvement in symptoms in the LFD group than in the control group (OR, 2.14; 95% CI, 1.56, 2.93). 

This study also explored the effect of LFD using IBS-SSS subscale scores and found that scores for four of the five questions showed a reduction in IBS symptom severity. Only “Interference with quality of life” did not show a significant improvement in the LFD group compared with the control group (SMD, −0.17; 95% CI, −0.40, 0.06). This result was somewhat relevant and consistent with the IBS-QoL analyses results, where the LFD group exhibited a slightly greater improvement in IBS-QoL than did the control group (SMD, 0.24; 95% CI, 0.02, 0.47). The reason behind this small effect size (SMD, 0.24) was probably the “food avoidance” subscale score, which showed a negative value of SMD, although it was assessed in four studies and had a broad 95% CI (SMD, −0.21; 95% CI, −0.44, 0.02; Figure S6). 

Three studies on stool habit change in patients with IBS-D showed a significant decrease in stool frequency (MD, −5.56/week; 95% CI, −7.40, −3.72) and a significant improvement in stool consistency (MD, −0.86; 95% CI, −1.52, −0.19) in the LFD group compared to that in the control group. This study result is consistent with those of previous studies that showed LFD is particularly effective at improving stool habits in IBS-D type patients [42,43]. This can be explained by the fact that food-related symptoms are most often reported by patients with IBS-D, and LFD induces a reduction in osmotic action exerted by FODMAPs in the intestinal lumen and reduces diarrhea [40]. Three other studies that evaluated the efficacy of LFD on stool habits included patients with all IBS subtypes except IBS-C, which made the MD value difficult to interpret clinically. Therefore, research using weighted stool scores that provides an estimate of how much the stool forms deviate from the optimal stool form [44] is needed. 

In the subgroup analyses, we found that the demonstrated improvements in IBS symptom severity were consistent between subgroups with different proportions of female participants, IBS subtypes, study durations and control interventions. The effect of study duration on IBS symptom reduction did not differ between 3–4 weeks and 6–12 weeks of intervention. When considering difficulties in following LFD compared to that of other treatments and the impact of food avoidance on quality of life [45], the initial rigorous dietary “eliminated” phase should be followed by a “reintroduction” phase within 3–6 weeks. The goal of the “reintroduction” phase is to find out which FODMAP groups trigger IBS symptoms and to improve the quality of life by allowing IBS patients to intake more food gradually. [46,47]. As expected, the effect of LFD on IBS symptom reduction was significantly greater when compared to the control group in the order of high-FODMAP diet, habitual diet, and dietary intervention (SMD, −0.95, −0.72, and −0.49, respectively). The four studies that compared LFD with other treatments (supplementation and lifestyle change) showed substantial between-study heterogeneity and a broad 95% CI magnitude of effect (SMD, −0.34; 95% CI −0.71, 0.03; I^2^ = 52%). 

According to a recent survey of over 155 United States gastroenterologists, the majority of surveyed gastroenterologists found LFD (84%), a lactose-free diet (65%), and a gluten-free diet (57%) to be “very effective” or “somewhat effective” for their IBS patients [48]. Therefore, we included studies that set a lactose-free diet or gluten-free diet as the control intervention, and even after including them, we found that the effect of LFD on IBS symptom reduction was still significantly higher than that of other dietary interventions (SMD, −0.49; 95% CI, −0.64, −0.34; I^2^ = 0%). Some studies have shown that IBS patients prefer and adhere highly to a gluten-free diet over LFD [49]. However, symptoms provoked by gluten restriction may largely be secondary to the restriction of FODMAP [50,51]. Krieger et al. found that LFD tended to be more advantageous than a lactose-free diet because only LFD effectively reduced pain and bloating in IBS patients, and suggested that only IBS patients with concomitant lactose malabsorption should follow a lactose-free diet [20]. In general, probiotics have resulted in improvement in IBS symptoms compared to a placebo [52,53,54]. However, adequately powered RCTs are still required to better determine the IBS subgroup, probiotic type, dose, and treatment duration that are most efficacious, to compare the efficacy of probiotics with LFD, and to explore the combined effect of LFD and probiotics.

The present meta-analysis had some limitations. The lack of standardization among studies could have introduced some bias. The implementation of LFD varied across different geographical regions, and the proportion of female participants or patients with different types of IBS was variable. In addition, some outcome assessments were conducted using a poor or not well-explained methodology. Even though we conducted subgroup analyses, all the subgroups in the current meta-analysis were relatively small, and as such, the outcomes should be interpreted with caution. Furthermore, it should be noted that the binary endpoint used in this study might not effectively capture symptom reduction, indicating the need for studies with large sample sizes to detect statistically significant differences between groups. 

## 5. Conclusions

In conclusion, LFD reduced IBS symptom severity and improved quality of life in patients with IBS compared to a control intervention which included structured or traditional IBS diet. However, food avoidance and its impact on quality of life should be investigated in patients who adopt LFD. Stool habits, including frequency and consistency, also improved in patients with IBS-D. Whether LFD is the most effective treatment option should be confirmed by future high-volume and well-designed RCTs focusing on efficacy predictors, comparisons with the supplement or lifestyle change, and long-term effects including the “reintroduction phase”.

## Figures and Tables

**Table 1:** Characteristics of studies included in the meta-analysis.

	First Author, Year	Country	Study Design	Sample	Intervention	Control	Duration of Therapy	Outcome Measures	Risk of Bias
				1. Sample size, *n*	*N* ^a^	1. Intervention (*n*) ^a^		Symptoms	
				2. Age, mean [SD] (range)				Quality of life	
				3. Female,%				BSFS	
				4. Diagnostic criteria				Stool frequency	
				5. IBS subtype					
				6. Drop out					
1	Goyal, 2021 [19]	India	RCT, single-blind	1. 101	*n* = 51	Traditional dietary advice (*n* = 49)	4 weeks	Symptoms	Good
				2. 41.9 [17.1]				QoL	
				3. 42%				BSFS	
				4. Rome IV				Stool frequency	
				5. IBS-D					
				6. 1					
2	Krieger, 2020 [20]	Switzer-land	RCT, cross-over	1. 29	*n* = 22	Low-lactose diet (*n* = 24)	3 weeks	Symptoms	Fair
				2. 30 (18–62)					
				3. 89.7%				BSFS	
				4. Rome IV					
				5. NA					
				6. 5					
3	Wilson, 2020 [21]	United Kingdom	RCT	1. 45	*n* = 22	Sham diet (*n* = 23)	4 weeks	Symptoms	Good
				2. 38.9 [10.0]				QoL	
				3. 55.1%					
				4. Rome III				Stool frequency	
				5. NA					
				6. 6					
4	Guerreiro, 2020 [22]	Portugal	Non-RCT	1. 70	*n* = 47	NICE guidelines (*n* = 23)	4 weeks	Symptoms	Good
				2. 48.5 [14.7]					
				3. 74.3%					
				4. Rome IV					
				5. All types; IBS-D (42.1%)					
				6. 13					
5	Catinean, 2019 [23]	Romania	RCT	1. 60	*n* = 30	Nutraceutical agent (*n* = 30)	24 days	Symptoms	Weak
				2. 40.37 [11.95]					
				3. 55%					
				4. Rome III					
				5. Excluding IBS-C					
				6. NR					
6	Paduano, 2019 [24]	Italy	Non-RCT, consecutive controlled study	1. 42	*n* = 34	Gluten-free (*n* = 30), Balanced Mediterranean diet (*n* = 28)	4 weeks	Symptoms	Fair
				2. 28.62 [6.86]				QoL	
				3. 83.3%					
				4. Rome IV					
				5, All type; IBS-D (52.4%)					
				6. 14					
7	Patcharatrakul, 2019 [25]	Thailand	RCT, single-blind	1. 66	*n* = 30	Brief advice on a commonly recommended diet (*n* = 32)	4 weeks	Symptoms	Fair
				2. 50 [13.7]					
				3. 76.7%					
				4. Rome III					
				5. All types; IBS-C (51.6%)					
				6. 4					
8	Zahedi, 2018 [26]	Iran	RCT, single-blind	1. 110	*n* = 50	General dietary advice in BDA (*n* = 51)	6 weeks	Symptoms	Good
				2. 37.6 [11.09]				QoL	
				3. 50.5%				BSFS	
				4. Rome II				Stool frequency	
				5. IBS-D					
				6. 9					
9	Schumann, 2018 [27]	Germany	RCT, single-blind	1. 59	*n* = 25	Yoga (*n* = 27)	12 weeks	Symptoms	Good
				2. 56.33 [9.5]				QoL	
				3. 88.1%					
				4. Rome III					
				5. All types; IBS-D (44.1%)					
				6. 7					
10	Pirkola, 2018 [28]	Finland	RCT, cross-over	1. 9	*n* = 7	Regular rye bread (*n* = 7)	1 day	Symptoms	Good
				2. 39 (29–51)					
				3. 100%					
				4. Rome III					
				5. All types; IBS-D (42.9%)					
				6. 2					
11	Eswaran, 2017 [29]	United States of America	RCT	1. 91	*n* = 45	Modified NICE guidelines (*n* = 39)	4 weeks	QoL	Good
				2. 42.6 (19–75)					
				3. 71%					
				4. Rome III					
				5. IBS-D					
				6. 7					
12	Harvie, 2017 [30]	New Zealand	RCT, non-blind	1. 50	*n* = 20	No dietary education (*n* = 25)	3 months	Symptoms	Good
				2. 43.3 [13.8]				QoL	
				3. 86%				BSFS	
				4. Rome III				Stool frequency	
				5. All types; IBS-D (62%)					
				6. 5					
13	Staudacher, 2017 [31]	Unitied Kingdom	RCT, 2 × 2 factorial design	1. 104	*n* = 51	Sham diet (*n* = 53)	4 weeks	Symptoms	Good
				2. 36 [11]				QoL	
				3. 67.3%				BSFS	
				4. Rome III				Stool frequency	
				5. Excluding IBS-C; IBS-D (66.3%)					
				6. 17					
14	McIntosh, 2017 [32]	Canada	RCT, single-blind	1. 40	*n* = 19	High-FODMAP (*n* = 18)	3 weeks	Symptoms	Good
				2. 50.28 (26–77)					
				3. 59.5%					
				4. Rome III					
				5. All types; IBS-D (27.8%)					
				6. 3					
15	Laatikainen, 2016 [33]	United States of America	RCT, cross-over	1. 80	*n* = 37	Traditional rye bread (*n* = 36)	4 weeks	Symptoms	Good
				2. 42.9 (21–64)				QoL	
				3. 91.3%					
				4. Rome III					
				5. Excluding IBS-C					
				6. 7					
16	Eswaran, 2016 [34]	United States of America	RCT	1. 92	*n* = 43	Modified NICE Guidelines (*n* = 39)	4 weeks	Symptoms	Good
				2. 42.6 (19–75)					
				3. 71%				BSFS	
				4. Rome III				Stool frequency	
				5. IBS-D					
				6. 8					
17	Peters, 2016 [35]	Australia	RCT, non-blind	1. 39	*n* = 21	Gut-directed hypnotherapy (*n* = 18)	6 weeks	Symptoms	Good
				2. 34 (23–66)				QoL	
				3. 81.1%					
				4. Rome III					
				5. All types; IBS-D (40.5%)					
				6. 12					
18	Böhn, 2015 [36]	Sweden	RCT, single-blind	1. 75	*n* = 33	NICE and the BDA guidelines (*n* = 34)	4 weeks	Symptoms	Good
				2. 43 [16]					
				3. 83.6%				BSFS	
				4. Rome III					
				5. All types; IBS-D (24%)					
				6. 8					
19	Pedersen, 2014 [37]	Denmark	RCT, non-blind	1. 123	*n* = 34	Probiotic Lactobacillus rhamnosus GG (*n* = 37), Normal Danish/Western diet (*n* = 37)	6 weeks	Symptoms	Good
				2. 37 (18–74)				QoL	
				3. 73%					
				4. Rome III					
				5. All types; IBS-D (40.7%)					
				6.15					
20	Halmos, 2014 [38]	Australia	RCT, cross-over	1. 33	*n* = 30	Typical Australian diet (*n* = 30)	3 weeks	Symptoms	Good
				2. 41 (29–53)					
				3. 70%					
				4. Rome III					
				5. All types; IBS-D (33.3%)					
				6. 3					
21	Staudacher, 2011 [39]	United Kingdom	RCT	1. 41	*n* = 16	Habitual diet (*n* = 19)	4 weeks	Symptoms	Good
				2. 35.2 [11.4]					
				3. 65.9%				BSFS	
				4. Rome III				Stool frequency	
				5. Excluding IBS-C					
				6. 6					
22	Ong, 2010 [40]	Australia	RCT, single-blind, cross-over	1. 15	*n* = 15	High-FODMAP diet (*n* = 15)	2 days	Symptoms	Good
				2. 41 (22–59)					
				3. 86.7%					
				4. Rome III					
				5. All types; IBS-D (26.7%)					
				6. NR					

BDA, British Dietetic Association; BSFS, Bristol stool form score; NA, not assessed; NICE, National Institute for Health and Care Excellence; NR, not reported; RCT, randomized controlled trial; QoL, quality of life; SD, standard deviation. ^a^ Numbers are retrieved from per-protocol data

**Table 2:** Results of subgroup analyses for different covariates.

Subgroup by	No. of Study	SMD (95% CI)	*P* for Heterogeneity	I^2^ (%)	*P* for between-Subgroup Heterogeniety
Female proportion					0.08
>75% (above median)	10	−0.39 (−0.63, −0.14)	0.05	46%	
<75% (below median)	10	−0.64 (−0.79, −0.49)	0.42	2%	
IBS subtype					0.65
All types	12	−0.56 (−0.80, −0.32)	0.01	54%	
Except IBS-C type	5	−0.43 (−0.70, −0.15)	0.22	31%	
IBS-D type	3	−0.60 (−0.84, −0.36)	0.73	0%	
Study duration					0.93
3–4 weeks	13	−0.51 (−0.68, −0.34)	0.13	32%	
6–12 weeks	5	−0.58 (−0.97, −0.19)	0.02	66%	
1–2 days	2	−0.44 (−1.18, 0.29)	0.24	28%	
Symptom assessment method					0.51
IBS-SSS	13	−0.49 (−0.66, −0.33)	0.11	34%	
Others	7	−0.62 (−0.94, −0.29)	0.06	50%	
Control group					0.23
Dietary intervention	9	−0.49 (−0.64, −0.34)	0.63	0%	
Habitual diet	5	−0.72 (−1.24, −0.2)	0.01	69%	
Other treatment	4	−0.34 (−0.71, 0.03)	0.1	52%	
High-FODMAP diet	2	−0.95 (−1.46, −0.44)	0.45	0%	

SMD, standardized mean difference.

**Table 3:** Risk of bias.

First Author, Publication Year	Random Sequence Generation	Allocation Conceal-Ment	Blinding of Participants and Personnel	Blinding of Outcome Assessment	Incomplete Outcome Data	Selective Reporting	Overall Quality
Goyal, 2021	Low	Low	High	Low	Low	Low	Good
Krieger, 2020	Low	Unclear	High	High	High	Low	Fair
Wilson, 2020	Low	Low	Unclear	Unclear	Low	Low	Good
Guerreiro, 2020	High	Low	High	Unclear	Low	Low	Good
Catinean, 2019	Unclear	Unclear	High	High	Unclear	Low	Weak
Paduano, 2019	High	Low	High	High	High	Low	Fair
Patcharatrakul, 2019	Unclear	Unclear	Unclear	Unclear	Low	Low	Fair
Zahedi, 2018	Low	Unclear	Low	High	Low	Low	Good
Schumann, 2018	Low	Low	High	Low	Low	Low	Good
Pirkola, 2018	Low	Unclear	Low	Low	Low	Low	Good
Eswaran, 2017	Low	Unclear	Unclear	Low	Low	Low	Good
Harvie, 2017	Low	Unclear	High	High	Low	Low	Good
Staudacher, 2017	Low	Low	Low	Low	Low	Low	Good
McIntosh, 2017	Low	Low	Unclear	Unclear	Low	Low	Good
Laatikainen, 2016	Low	Unclear	Low	Low	Low	High	Good
Eswaran, 2016	Low	Unclear	Unclear	Low	Low	Low	Good
Peters, 2016	Low	Unclear	High	High	Low	Low	Good
Böhn, 2015	Low	Low	Low	High	Low	Low	Good
Pedersen, 2014	Low	Unclear	High	High	Low	Low	Good
Halmos, 2014	Low	Unclear	Low	Unclear	Low	Low	Good
Staudacher, 2012	Low	Low	Unclear	Unclear	Low	Low	Good
Ong, 2010	Low	Unclear	Low	High	Low	Low	Good

## Data Availability

Data are available upon request.

## Supplementary Materials

The following are available online at https://www.mdpi.com/article/10.3390/nu13072460/s1: Figure S1—Forest plot for subgroup analysis of female proportion; Figure S2—Forest plot for subgroup analysis of IBS subtype; Figure S3—Forest plot for subgroup analysis of study duration; Figure S4—Forest plot for subgroup analysis of symptom assessment method; Figure S5—Forest plot for subgroup analysis of control group type; Figure S6—Forest plot showing standardized mean differences for IBS-QoL subscale score; Table S1—Sensitivity analysis for the outcome of IBS symptoms severity; Table S2—Sensitivity analysis for the outcome of IBS-related quality of life.
