# Supplementary material for: Effect of Low FODMAPs Diet on Irritable Bowel Syndromes: A Systematic Review and Meta-Analysis of Clinical Trials
Source: Nutrients. 2021 Jul 19;13(7):2460. doi: 10.3390/nu13072460 (PMC8308820; doi:10.3390/nu13072460)
Supplement: Supplementary file 1 [file nutrients-13-02460-s001.zip › nutrients-1290279-SI.pdf]

Study	Experimental Total	Experimental Mean	Experimental SD	Control Total	Control Mean	Control SD	SMD	95%-CI	Weight
**Female_proportions = <75%**									
Goyal, 2021	51	-135.20	115.80	49	-81.40	111.52	-0.47	[-0.87; -0.07]	6.9%
Wilson, 2020	22	183.80	89.59	23	217.10	88.72	-0.37	[-0.96; 0.22]	4.3%
Catinean, 2019	30	-35.16	42.17	30	-23.83	44.52	-0.26	[-0.77; 0.25]	5.3%
Zahedi, 2018	50	-155.75	81.09	51	-102.75	74.56	-0.68	[-1.08; -0.27]	6.8%
Staudacher, 2017	51	173.00	95.00	53	224.00	89.00	-0.55	[-0.94; -0.16]	7.0%
McIntosh, 2017	19	-81.60	78.28	18	19.00	95.69	-1.13	[-1.83; -0.43]	3.4%
Eswaran , 2016	43	-1.83	1.80	39	-0.60	1.92	-0.66	[-1.10; -0.21]	6.1%
Pedersen, 2014	34	-133.00	122.00	74	-51.00	101.93	-0.75	[-1.17; -0.33]	6.5%
Halmos, 2014	30	73.10	53.10	30	137.00	74.05	-0.98	[-1.52; -0.44]	4.9%
Staudacher, 2012	16	1.10	0.51	19	1.70	0.56	-1.09	[-1.81; -0.38]	3.3%
**Random effects model**	**346**			**386**			**-0.64**	**[-0.79; -0.49]**	**54.4%**
Heterogeneity: $I^2 = 2\%$, $\tau^2 = 0.0010$, $p = 0.42$									
**Female_proportions = >75%**									
Krieger, 2020	22	-87.75	97.14	24	-33.85	109.30	-0.51	[-1.10; 0.08]	4.4%
Paduano, 2019	34	16.00	8.00	58	18.07	8.12	-0.25	[-0.68; 0.17]	6.4%
Patcharatrakul, 2019	30	38.50	20.00	32	53.50	19.20	-0.76	[-1.27; -0.24]	5.2%
Schumann, 2018	25	-96.18	88.30	27	-66.16	80.91	-0.35	[-0.90; 0.20]	4.8%
Pirkola, 2018	7	8.25	10.33	7	7.90	7.99	0.04	[-1.01; 1.08]	1.8%
Harvie, 2017	20	-144.50	89.00	25	-38.70	74.80	-1.28	[-1.93; -0.63]	3.8%
Laatikainen, 2016	37	199.00	62.10	36	207.00	61.20	-0.13	[-0.59; 0.33]	5.9%
Peters, 2016	21	-30.00	26.89	28	-33.00	21.60	0.12	[-0.44; 0.69]	4.6%
Bohn, 2015	33	-77.00	110.00	34	-65.00	84.00	-0.12	[-0.60; 0.36]	5.6%
Ong, 2010	15	0.67	0.13	15	1.60	1.73	-0.74	[-1.48; 0.00]	3.1%
**Random effects model**	**244**			**286**			**-0.39**	**[-0.63; -0.14]**	**45.6%**
Heterogeneity: $I^2 = 46\%$, $\tau^2 = 0.0689$, $p = 0.05$									
**Random effects model**	**590**			**672**			**-0.53**	**[-0.68; -0.38]**	**100.0%**
Heterogeneity: $I^2 = 39\%$, $\tau^2 = 0.0445$, $p = 0.04$									
Test for subgroup differences: $\chi^2_1 = 3.02$, df = 1 ($p = 0.08$)									

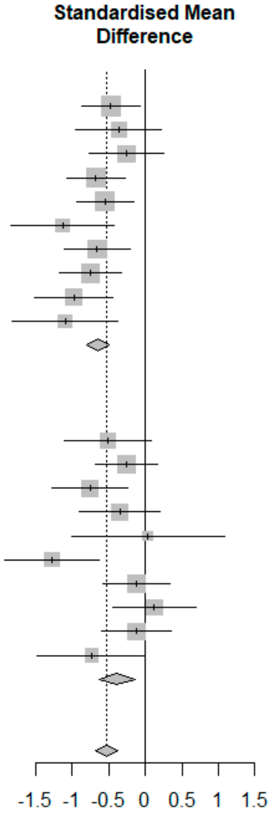

Figure S1. Forest plot for subgroup analysis on female proportion

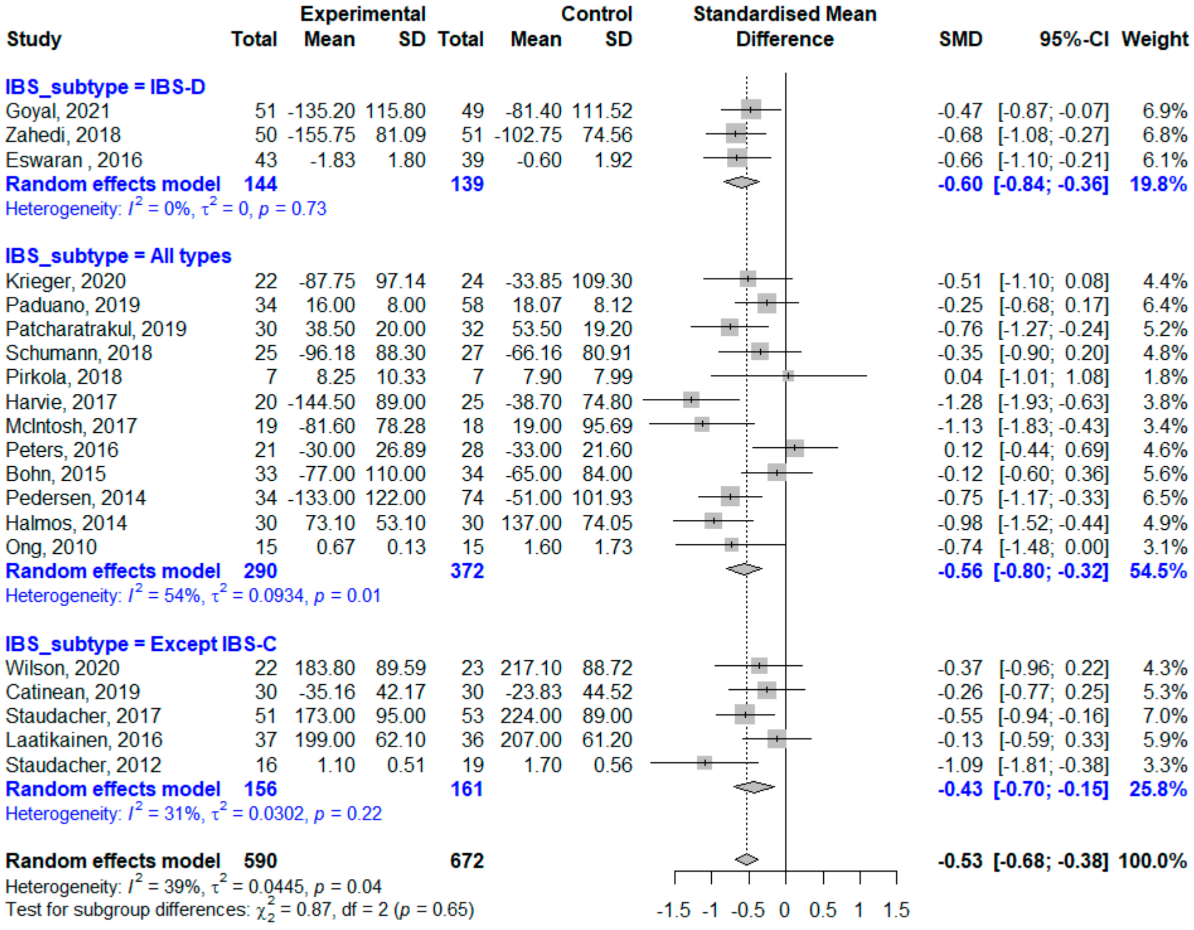

Figure S2. Forest plot for subgroup analysis on IBS subtype

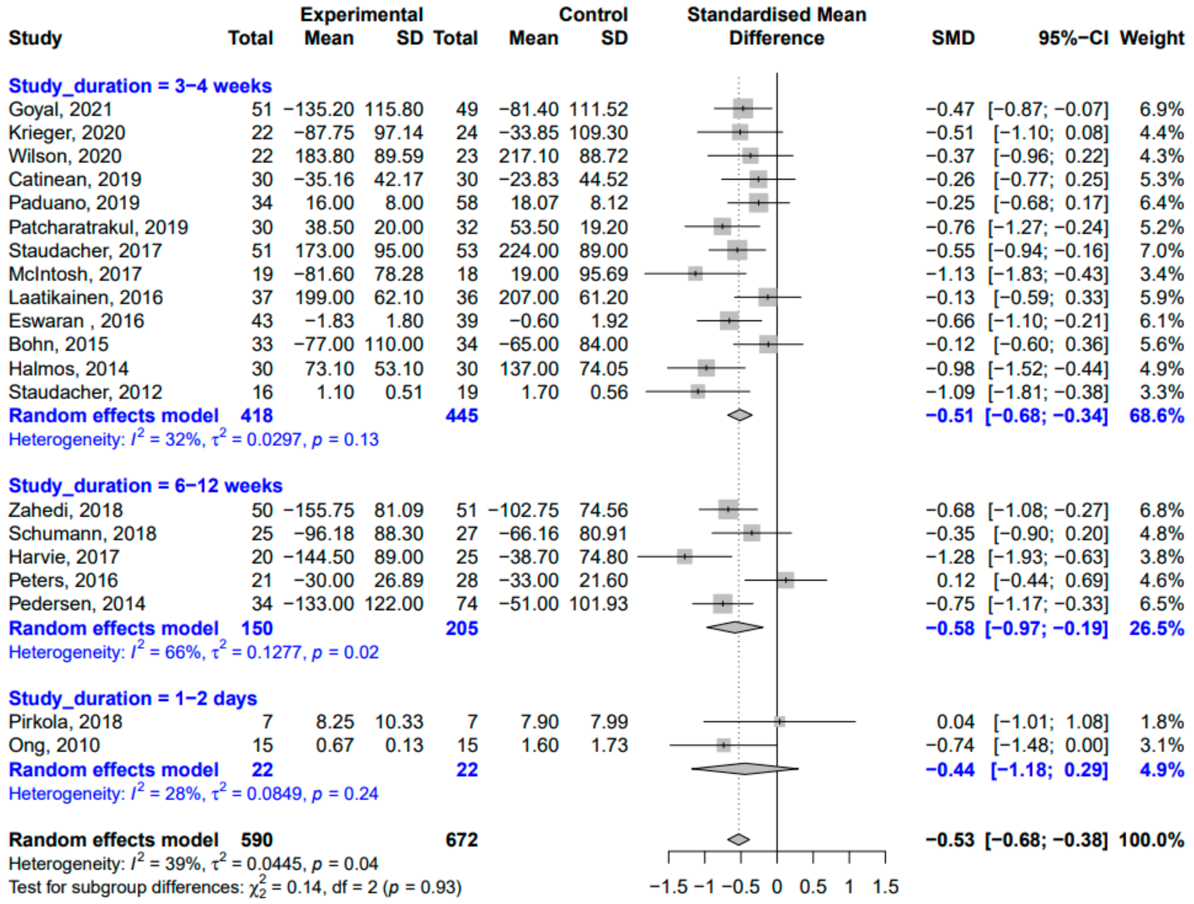

Figure S3. Forest plot for subgroup analysis on study duration

Study	Experimental Total	Experimental Mean	Experimental SD	Control Total	Control Mean	Control SD	SMD	95%−CI	Weight
**Symptom_assessment_method = IBS−SSS**									
Goyal, 2021	51	−135.20	115.80	49	−81.40	111.52	−0.47	[−0.87; −0.07]	6.9%
Krieger, 2020	22	−87.75	97.14	24	−33.85	109.30	−0.51	[−1.10; 0.08]	4.4%
Wilson, 2020	22	183.80	89.59	23	217.10	88.72	−0.37	[−0.96; 0.22]	4.3%
Catinean, 2019	30	−35.16	42.17	30	−23.83	44.52	−0.26	[−0.77; 0.25]	5.3%
Paduano, 2019	34	16.00	8.00	58	18.07	8.12	−0.25	[−0.68; 0.17]	6.4%
Zahedi, 2018	50	−155.75	81.09	51	−102.75	74.56	−0.68	[−1.08; −0.27]	6.8%
Schumann, 2018	25	−96.18	88.30	27	−66.16	80.91	−0.35	[−0.90; 0.20]	4.8%
Harvie, 2017	20	−144.50	89.00	25	−38.70	74.80	−1.28	[−1.93; −0.63]	3.8%
Staudacher, 2017	51	173.00	95.00	53	224.00	89.00	−0.55	[−0.94; −0.16]	7.0%
McIntosh, 2017	19	−81.60	78.28	18	19.00	95.69	−1.13	[−1.83; −0.43]	3.4%
Laatikainen, 2016	37	199.00	62.10	36	207.00	61.20	−0.13	[−0.59; 0.33]	5.9%
Bohn, 2015	33	−77.00	110.00	34	−65.00	84.00	−0.12	[−0.60; 0.36]	5.6%
Pedersen, 2014	34	−133.00	122.00	74	−51.00	101.93	−0.75	[−1.17; −0.33]	6.5%
**Random effects model**	**428**			**502**			**−0.49**	**[−0.66; −0.33]**	**71.1%**
Heterogeneity: $I^2 = 34\%$, $\tau^2 = 0.0316$, $p = 0.11$									
**Symptom_assessment_method = Others**									
Patcharatrakul, 2019	30	38.50	20.00	32	53.50	19.20	−0.76	[−1.27; −0.24]	5.2%
Pirkola, 2018	7	8.25	10.33	7	7.90	7.99	0.04	[−1.01; 1.08]	1.8%
Eswaran , 2016	43	−1.83	1.80	39	−0.60	1.92	−0.66	[−1.10; −0.21]	6.1%
Peters, 2016	21	−30.00	26.89	28	−33.00	21.60	0.12	[−0.44; 0.69]	4.6%
Halmos, 2014	30	73.10	53.10	30	137.00	74.05	−0.98	[−1.52; −0.44]	4.9%
Staudacher, 2012	16	1.10	0.51	19	1.70	0.56	−1.09	[−1.81; −0.38]	3.3%
Ong, 2010	15	0.67	0.13	15	1.60	1.73	−0.74	[−1.48; 0.00]	3.1%
**Random effects model**	**162**			**170**			**−0.62**	**[−0.94; −0.29]**	**28.9%**
Heterogeneity: $I^2 = 50\%$, $\tau^2 = 0.0925$, $p = 0.06$									
**Random effects model**	**590**			**672**			**−0.53**	**[−0.68; −0.38]**	**100.0%**
Heterogeneity: $I^2 = 39\%$, $\tau^2 = 0.0445$, $p = 0.04$									
Test for subgroup differences: $\chi^2_1 = 0.44$, df = 1 ($p = 0.51$)									

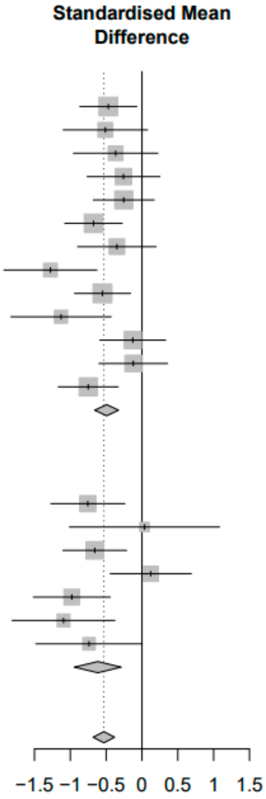

Figure S4. Forest plot for subgroup analysis on symptom assessment method

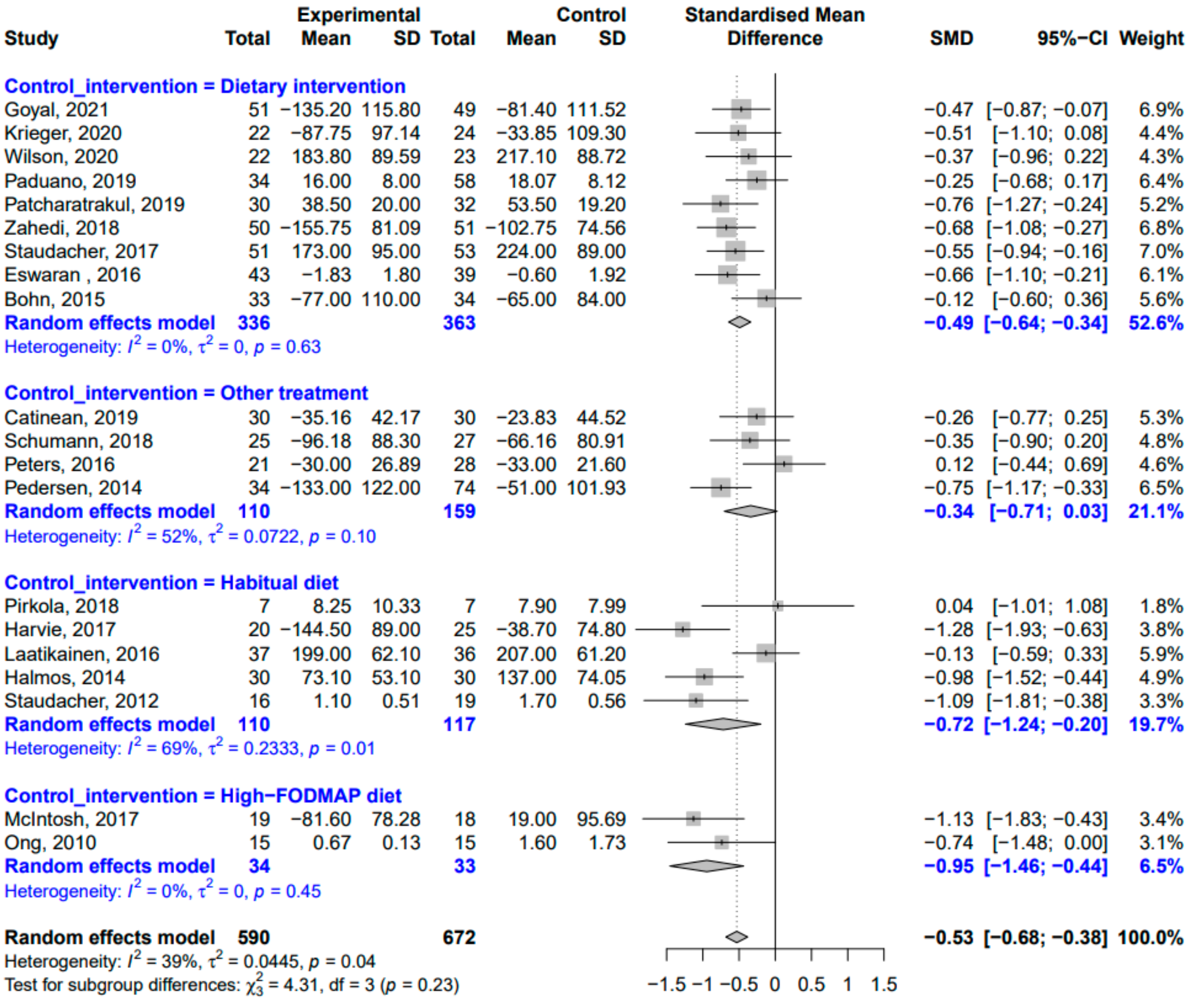

Figure S5. Forest plot for subgroup analysis on control group type

Study	Experimental Total	Experimental Mean	Experimental SD	Control Total	Control Mean	Control SD	SMD	95%-CI	Weight
**Dysphoria**									
Wilson,2020	22	75.50	16.89	23	70.30	16.79	0.30	[−0.28; 0.89]	2.0%
Paduano, 2019	34	86.00	13.00	58	81.48	12.54	0.35	[−0.07; 0.78]	3.7%
Zahedi, 2018	25	7.71	16.76	27	0.21	18.38	0.42	[−0.13; 0.97]	2.2%
Staudacher, 2017	51	71.90	24.70	53	72.20	20.50	−0.01	[−0.40; 0.37]	4.6%
**Random effects model**	**132**			**161**			**0.22**	**[−0.01; 0.46]**	**12.5%**
Heterogeneity: $I^2 = 0\%$, $\tau^2 = 0$, $p = 0.50$									
**Interference with activity**									
Wilson, 2020	22	71.20	16.42	23	71.80	16.79	−0.04	[−0.62; 0.55]	2.0%
Paduano, 2019	34	80.00	19.00	58	77.45	18.51	0.14	[−0.29; 0.56]	3.8%
Schumann, 2018	25	7.89	16.03	27	−1.27	13.43	0.61	[0.05; 1.17]	2.2%
Staudacher, 2017	51	72.90	24.20	53	71.20	20.60	0.08	[−0.31; 0.46]	4.6%
**Random effects model**	**132**			**161**			**0.17**	**[−0.06; 0.40]**	**12.5%**
Heterogeneity: $I^2 = 5\%$, $\tau^2 = 0$, $p = 0.37$									
**Body image**									
Wilson, 2020	22	72.00	15.48	23	69.20	15.83	0.18	[−0.41; 0.76]	2.0%
Paduano, 2019	34	77.00	16.00	58	76.45	14.03	0.04	[−0.39; 0.46]	3.8%
Schumann, 2018	25	8.04	16.22	27	−1.57	20.29	0.51	[−0.04; 1.07]	2.2%
Staudacher, 2017	51	73.20	22.70	53	64.20	22.70	0.39	[0.01; 0.78]	4.5%
**Random effects model**	**132**			**161**			**0.27**	**[0.04; 0.51]**	**12.4%**
Heterogeneity: $I^2 = 0\%$, $\tau^2 = 0$, $p = 0.50$									
**Health worry**									
Wilson, 2020	22	75.20	16.89	23	68.50	17.26	0.39	[−0.21; 0.98]	1.9%
Paduano, 2019	34	82.00	17.00	58	82.00	14.09	0.00	[−0.42; 0.42]	3.8%
Schumann, 2018	25	10.24	16.41	27	−3.01	19.04	0.73	[0.17; 1.30]	2.1%
Staudacher, 2017	51	73.00	20.00	53	71.10	20.80	0.09	[−0.29; 0.48]	4.6%
**Random effects model**	**132**			**161**			**0.22**	**[−0.01; 0.45]**	**12.4%**
Heterogeneity: $I^2 = 39\%$, $\tau^2 = 0$, $p = 0.18$									
**Food avoidance**									
Wilson, 2020	22	56.80	20.17	23	62.70	20.14	−0.29	[−0.88; 0.30]	2.0%
Paduano, 2019	34	64.00	29.00	58	62.93	26.00	0.04	[−0.38; 0.46]	3.8%
Schumann, 2018	25	−10.89	26.50	27	2.17	25.62	−0.49	[−1.05; 0.06]	2.2%
Staudacher, 2017	51	51.10	26.70	53	57.90	29.20	−0.24	[−0.63; 0.14]	4.5%
**Random effects model**	**132**			**161**			**−0.21**	**[−0.44; 0.02]**	**12.5%**
Heterogeneity: $I^2 = 0\%$, $\tau^2 = 0$, $p = 0.49$									
**Social relation**									
Wilson, 2020	22	72.60	16.89	23	73.00	16.79	−0.02	[−0.61; 0.56]	2.0%
Paduano, 2019	34	87.00	12.00	58	85.97	12.57	0.08	[−0.34; 0.51]	3.8%
Schumann, 2018	25	4.69	15.42	27	5.27	19.24	−0.03	[−0.58; 0.51]	2.3%
Staudacher, 2017	51	77.50	22.40	53	71.70	22.20	0.26	[−0.13; 0.64]	4.5%
**Random effects model**	**132**			**161**			**0.11**	**[−0.12; 0.34]**	**12.6%**
Heterogeneity: $I^2 = 0\%$, $\tau^2 = 0$, $p = 0.79$									
**Sexual issue**									
Wilson, 2020	22	90.20	12.20	23	86.20	11.99	0.33	[−0.26; 0.91]	2.0%
Paduano, 2019	34	93.00	11.00	58	93.93	12.30	−0.08	[−0.50; 0.35]	3.8%
Schumann, 2018	25	1.02	23.78	27	8.60	27.07	−0.29	[−0.84; 0.25]	2.3%
Staudacher, 2017	51	79.70	24.70	53	76.20	28.60	0.13	[−0.26; 0.51]	4.6%
**Random effects model**	**132**			**161**			**0.02**	**[−0.21; 0.25]**	**12.5%**
Heterogeneity: $I^2 = 0\%$, $\tau^2 = 0$, $p = 0.42$									
**Relationship issue**									
Wilson, 2020	22	80.70	15.48	23	79.00	15.35	0.11	[−0.48; 0.69]	2.0%
Paduano, 2019	34	88.00	15.00	58	86.52	15.36	0.10	[−0.33; 0.52]	3.8%
Schumann, 2018	25	2.90	17.36	27	1.39	267.18	0.01	[−0.54; 0.55]	2.3%
Staudacher, 2017	51	81.20	18.80	53	80.50	19.90	0.04	[−0.35; 0.42]	4.6%
**Random effects model**	**132**			**161**			**0.06**	**[−0.17; 0.29]**	**12.6%**
Heterogeneity: $I^2 = 0\%$, $\tau^2 = 0$, $p = 0.99$									
**Random effects model**	**1056**			**1288**			**0.11**	**[0.03; 0.19]**	**100.0%**

Heterogeneity: $I^2 = 0\%$, $\tau^2 < 0.0001$, $p = 0.47$
Test for subgroup differences: $\chi^2_7 = 11.78$, df = 7 ($p = 0.11$)

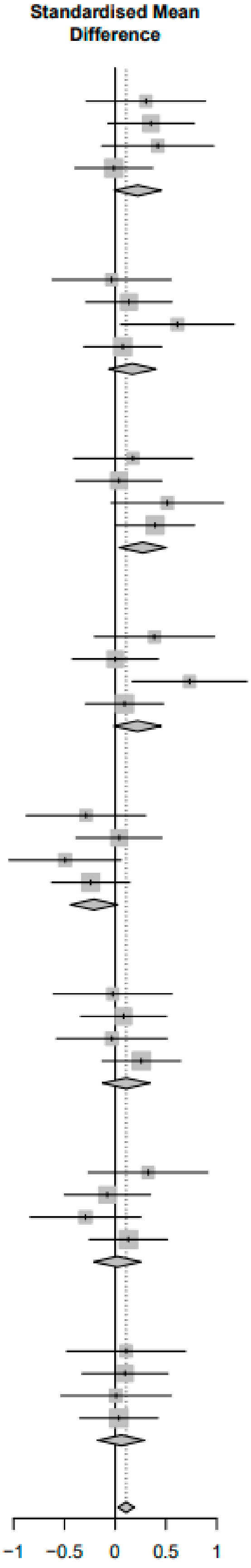

Figure S6. Forest plot showing standardized mean differences for IBS-QoL subscale score

Table S1. Sensitivity analysis for the outcome of IBS symptoms severity

Omitting	SMD [95% CI]
Goyal, 2021	0.528 [-0.647; -0.409]
Krieger, 2020	0.524[-0.640; -0.407]
Wilson, 2020	0.529 [-0.646; -0.413]
Catinean, 2019	0.537 [-0.655; -0.420]
Paduano, 2019	0.544 [-0.663; -0.426]
Patcharatrakul, 2019	0.511 [-0.628; -0.394]
Zahedi, 2018	0.51 [-0.629; -0.391]
Schumann, 2018	0.531 [-0.648; -0.414]
Pirkola, 2018	0.53 [-0.645; -0.415]
Harvie, 2017	0.499 [-0.615; -0.383]
Staudacher, 2017	0.521 [-0.640; -0.401]
McIntosh, 2017	0.507 [-0.622; -0.391]
Laatikainen, 2016	0.549 [-0.667; -0.431]
Eswaran, 2016	0.514 [-0.632; -0.396]
Peters, 2016	0.551 [-0.667; -0.434]
Bohn, 2015	0.547 [-0.665; -0.430]
Pedersen, 2014	0.505 [-0.624; -0.386]
Halmos, 2014	0.502 [-0.619; -0.385]
Staudacher, 2012	0.509 [-0.624; -0.393]
Ong, 2010	0.518 [-0.634; -0.403]
Goyal, 2021	0.528 [-0.647; -0.409]

Table S2. Sensitivity analysis for the outcome of IBS-related quality of life

Omitting	SMD (95% CI)
Goyal, 2021	0.223 [0.076; 0.371]
Wilson, 2020	0.185 [0.076; 0.371]
Paduano, 2019	0.211[0.065; 0.358]
Zahedi, 2018	0.275 [0.127; 0.423]
Schumann, 2018	0.203 [0.060; 0.346]
Eswaran, 2017	0.164 [0.019; 0.310]
Harvie, 2017	0.169 [0.027; 0.311]
Staudacher, 2017	0.231 [0.083; 0.380]
Laatikainen, 2016	0.241 [0.096; 0.386]
Peters, 2016	0.244 [0.102; 0.385]
Pedersen, 2014	0.207 [0.060; 0.354]
